# Monocyte to high-density lipoprotein ratio based prognostic nomogram for patients following allogeneic vascular replacement pancreaticoduodenectomy

**DOI:** 10.3389/fgene.2024.1465318

**Published:** 2024-08-26

**Authors:** Xiao-Wen Ye, Zu-Yu Wang, Yun-Xia Shao, Ying-Chun Tang, Xiong-Jun Dong, Ya-Ning Zhu

**Affiliations:** ^1^ Department of Nephrology, Wuhu Hospital, East China Normal University, Wuhu, China; ^2^ Department of Hepatobiliary and Pancreaticosplenic Surgery, Beijing Chaoyang Hospital, Capital Medical University, Beijing, China

**Keywords:** immune-inflammatory metabolic marker, monocyte to high-density lipoprotein ratio, creatinine, allogeneic vascular replacement, prognosis

## Abstract

**Background:**

Preoperative immune-inflammatory condition influencing the metabolism of malignancies. We sought to investigate the prognostic value of a novel immune-inflammatory metabolic marker, the monocyte-to-high-density lipoprotein ratio (MHR), in patients with locally advanced pancreatic cancer.

**Methods:**

A retrospective analysis was conducted on the clinical data of 118 patients with locally advanced pancreatic cancer and obstructive jaundice who underwent allogeneic vascular replacement pancreaticoduodenectomy in our hospital from Apr. 2011 to Dec. 2023. To assess the predictive capacity of immune-inflammatory metabolic marker, we utilized the area under the receiver operating characteristic curve (AUC-ROC) and assessed the predictive potential of MHR in forecasting outcomes through both univariate and multivariate Cox proportional hazard analyses.

**Results:**

The area under AUC for MHR in predicting 1-year postoperative survival was 0.714, with an optimal cutoff value of 1.184, yielding a sensitivity of 78.9% and specificity of 66.2%. Based on this cutoff value, patients were divided into a low MHR group (MHR ≤1.184, n = 61) and a high MHR group (MHR >1.184, n = 57). The median survival times for the low and high MHR groups were 27.0 months and 12.0 months, respectively (χ2 = 30.575, *p* < 0.001), and the median DFS were 18.0 months and 8.0 months, respectively (χ2 = 26.330, *p* < 0.001). Univariate and multivariate analyses indicated that preoperative MHR, preoperative creatinine, operation duration, and TNM stage were independent predictors of postoperative mortality, while preoperative MHR, preoperative creatinine, and TNM stage were independent predictors of postoperative recurrence risk.

**Conclusion:**

MHR, as an independent immune-inflammatory metabolic predictor of OS and DFS in patients with advanced PC after pancreaticoduodenectomy. Early monitoring and reduction of MHR may be of great significance in improving prognosis.

## Introduction

In recent years, the incidence and mortality rates of pancreatic cancer have shown a significant upward trend. As one of the malignancies with the poorest prognosis, the 5-year survival rate of pancreatic cancer is less than 10%, with 80% of patients already at an advanced stage or having distant metastasis at the time of diagnosis, thereby losing the opportunity for surgical intervention (Siegel et al., 2021; Huang et al., 2021; Wang et al., 2021a). Despite the significant advancements in chemotherapy and immune-targeted therapies for pancreatic cancer, radical surgery remains the only effective method for providing a chance of cure and long-term survival for patients (Zhou et al., 2021). However, even after radical surgical resection, the survival rate of pancreatic cancer patients remains low, and they are prone to early recurrence (Wang et al., 2021b). Therefore, there is a need for novel biomarkers to predict the risk of recurrence and mortality in local advanced pancreatic cancer patients undergoing radical resection and allogeneic vascular replacement surgery, in order to implement early interventions for high-risk patients and thereby improve prognosis and survival rates.

Inflammation is a key factor in the occurrence and development of tumors, and shorter survival times are closely related to systemic inflammation. Monocytes, as innate immune cells of the mononuclear phagocyte system, have become important regulators of cancer development and progression. Their peripheral blood levels can reflect the severity of inflammation in both the systemic and tumor microenvironments (Fang et al., 2021). Monocytes are recruited to the tumor site through CCL2/CCR2, inhibiting CD8^+^ T cell infiltration and recruiting regulatory T cells (Tregs). Tregs within the tumor can produce cytokines such as IL-4, IL-10, and IL-13, causing monocytes to differentiate into tumor-associated macrophages (TAMs) (Pushalkar et al., 2018). Pro-angiogenic monocytes and TAMs in the tumor microenvironment can also release angiogenesis-related growth factors and pro-inflammatory cytokines, inducing tumor angiogenesis and the epithelial-mesenchymal transition (EMT) process in pancreatic cancer (PC), thereby promoting tumor growth and metastasis (Chen et al., 2021). Biomarkers based on peripheral blood monocytes may signal the occurrence and development of tumors. Dyslipidemia is increasingly recognized as an important mechanism in tumor occurrence, with the metastatic ability of tumor cells being closely related to lipid metabolism enzymes (Cruz et al., 2013). High-density lipoprotein (HDL), as an important lipoprotein, is considered a protective factor against pancreatic cancer and has been shown to play a role in various tumors, including thyroid cancer, breast cancer, gastric cancer, and colorectal cancer, and is associated with the occurrence, progression, and poor prognosis of these diseases (Mazzuferi et al., 2021). HDL inhibits tumor growth through anti-inflammatory and antioxidant effects, inhibition of angiogenesis, regulation of signal transduction, and enhancement of anti-tumor immune responses (Ben-Aicha et al., 2020; Qin et al., 2023).

The monocyte to high-density lipoprotein ratio (MHR) is a novel inflammatory marker that has been shown to play a role in many tumors and is closely related to the occurrence, development, and prognosis of tumors (Xu et al., 2021; Wu et al., 2023; Liu et al., 2024; Miao et al., 2024). However, the impact of MHR on the prognosis of pancreatic cancer patients with vascular invasion undergoing radical resection and allogeneic vascular replacement surgery remains unclear. Therefore, the purpose of this study is to explore the correlation between MHR and the risk of death and tumor recurrence in pancreatic cancer patients undergoing radical resection and allogeneic vascular replacement surgery, and to establish a new predictive model based on MHR.

## Methods

### Ethics approval and consent to participate

The study was conducted in accordance with the Declaration of Helsinki (as revised in 2013) and approved by the Ethics Committee of Wuhu Hospital Affiliated to East China Normal University (No. 2021-D.-302) and the Ethics Committee of Beijing Chaoyang Hospital (No.2020-D.-309-2). Due to the retrospective nature of the study, participant informed consent was waived, and the study design was approved by the appropriate ethics review board.

### Patients and clinicopathological factors

#### Patient selection

Our study retrospectively analyzed the data of pancreatic cancer patients who underwent radical surgery combined with allogeneic vascular replacement in our hospital from Apr. 2011 to Dec. 2023 and screened out 118 eligible patients for further analysis according to the inclusion and exclusion criteria. The authors are accountable for all aspects of the work in ensuring that questions related to the accuracy or integrity of any part of the work are appropriately investigated and resolved.

#### Inclusion and exclusion criteria

Inclusion criteria: (Siegel et al., 2021): Patients diagnosed with pancreatic cancer and with vascular invasion at Beijing Chaoyang Hospital, Capital Medical University, during this period; (Huang et al., 2021); Patients who had not received any preoperative radiotherapy, chemotherapy, immunotherapy, or targeted therapy; (Wang et al., 2021a); Pathological confirmation of pancreatic cancer postoperatively; (Zhou et al., 2021); Patients and their families provided informed consent for the surgical procedure.

Exclusion criteria: (Siegel et al., 2021): Distant metastasis found during preoperative examinations or intraoperative exploration; (Huang et al., 2021); Perioperative mortality; (Wang et al., 2021a); Incomplete follow-up data; (Zhou et al., 2021); Severe comorbid conditions (e.g., heart disease, kidney disease) that could affect pancreatic cancer treatment or prognosis; (Wang et al., 2021b); Patients who did not undergo surgical treatment for pancreatic cancer.

### Patients grouping and definition

MHR was calculated from peripheral blood monocyte counts and high-density lipoprotein (HDL) levels obtained from routine blood tests and blood biochemistry conducted 1 week before surgery. MHR = total peripheral blood monocyte count (x10^9/L)/HDL (mmol/L). The receiver operating characteristic (ROC) curve was used to determine the optimal cutoff value of MHR for predicting 1-year postoperative survival, which was 1.184. Patients were divided into a high MHR group (n = 57, MHR >1.184) and a low MHR group (n = 61, MHR ≤1.184).

### Perioperative management

**FIGURE 1:**
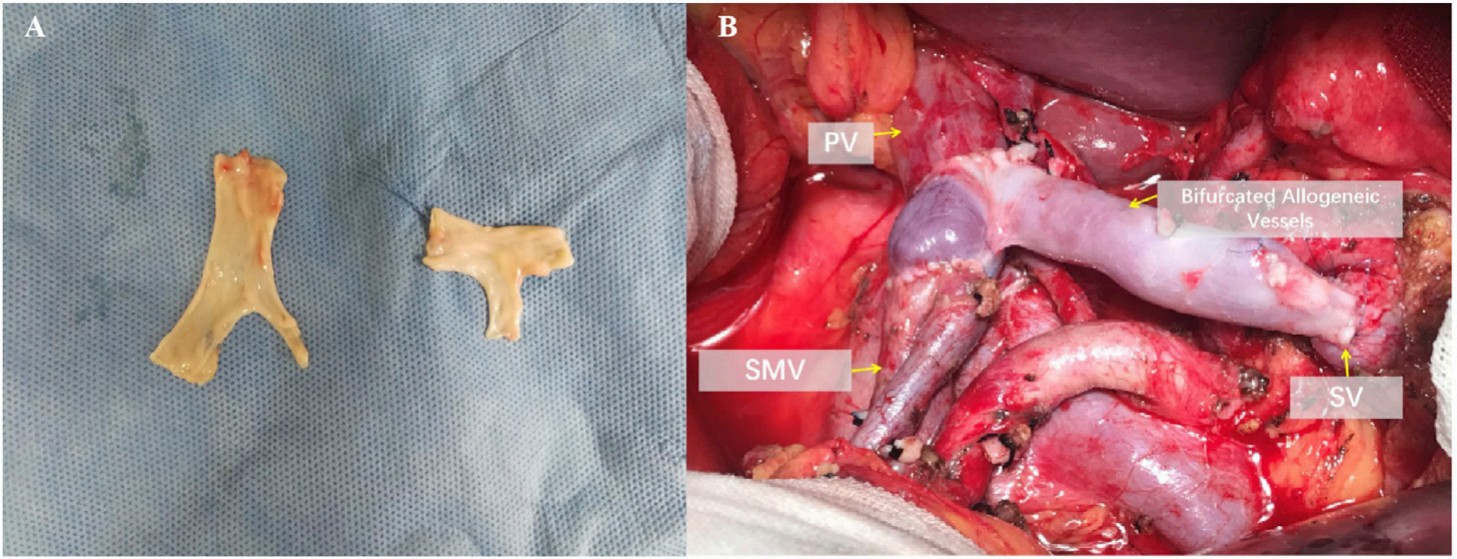
Reconstruction after resection of the portal vein system. **(A)** Intraoperative photograph of bifurcated allogeneic vein reconstruction of portal vein-superior mesenteric vein-splenic vein. **(B)** Preparation method and ex vivo morphology of bifurcated allogeneic vessels.

According to the Pancreatic Cancer Diagnosis and Treatment Guidelines (2022 edition), borderline resectable pancreatic cancer cases underwent allogeneic vascular replacement combined with radical surgery (Figure 1). Standard lymph node dissection for radical pancreatic cancer surgery was performed intraoperatively. Postoperative complications and subsequent chemotherapy were recorded. Pathological specimens were prepared as paraffin sections, and reports were provided by the pathology department.

### Surgical procedure of intraoperative vascular replacement

First, use vascular clamps to respectively block the PV (portal vein), SMV (superior mesenteric vein), and SV (splenic vein). Then, sever the vessels more than 5 mm from the tumor invasion margin, and excise the tumor along with the invaded vessels *en bloc*. Send the intraoperative frozen sections of the three severed vessels for pathological margin assessment. Meanwhile, take the cryopreserved allogeneic vessels and tailor them according to the vascular defect *ex vivo*. After confirming negative margins from the intraoperative pathology analysis, proceed with the reconstruction using the allogeneic vessels. To alleviate intestinal congestion and quickly restore intestinal blood flow, we prioritize the reconstruction of the SMV. Using 6-0 prolene sutures, perform continuous everting anastomosis of the anterior and posterior walls between the allogeneic vessel and the SMV. Then, perform similar anastomosis between the allogeneic vessel and the PV. Release the vascular clamps on the PV and SMV to restore blood flow between them. Subsequently, perform continuous everting anastomosis of the anterior and posterior walls between the allogeneic vessel and the SV using 6-0 prolene sutures. Release the clamp on the SV to restore blood flow between the SV and the PV, thus completing the allogeneic vessel replacement.

### Observational indicators and follow-up strategy

General patient data, routine blood tests, biochemical tests, tumor markers, imaging results, surgical-related indicators, vascular replacement status, and pathological results were collected. Postoperative follow-up combined outpatient review and telephone follow-up to monitor patient survival. Patients were reviewed at 1 and 3 months postoperatively, every 3 months for the first 2 years, and every 6 months thereafter. Follow-up assessments included routine blood tests, biochemical tests, tumor markers, and imaging examinations. Follow-up ended in Jan. 2024.

### Statistical analysis

Statistical analysis was performed using SPSS 26.0 software. Normally distributed continuous data were expressed as mean ± standard deviation (x ± s) and compared between groups using the independent samples *t*-test. Non-normally distributed continuous data were expressed as median (Q1, Q3) and compared between groups using the Mann-Whitney *U* test. Categorical data were expressed as frequencies (percentages), and comparisons between groups were made using the chi-square test with Bonferroni correction for multiple comparisons. Survival analysis was performed using the Kaplan-Meier method, with survival rates compared using the log-rank test. Factors with *p* < 0.1 in univariate analysis were included in the Cox proportional hazards model for multivariate analysis to identify prognostic factors, and results were expressed as hazard ratios (HR) with 95% confidence intervals (95% CI). A *p*-value <0.05 was considered statistically significant. The ROC curve was used to evaluate the predictive value of MHR for 1-year prognosis in PC patients after radical surgery. The area under the ROC curve (AUC) was used to assess the accuracy of the cutoff value.

### Construction and validation of the prognostic prediction model nomogram

Factors that reached statistical significance in univariate Cox regression analysis were included in the model to construct the prognostic prediction model nomogram. The performance of the nomogram was evaluated using receiver operating characteristic (ROC) curves (using the “pROC” package in R), bootstrapped c-index (using the “rms” package in R), and calibration plots (using the “rms” package in R). The benefit curve of the model was constructed using the “ggDCA” package.

### Quality control management

(1) Data collection: All research data was collected by highly trained professionals. They followed predetermined procedures to extract information from the electronic medical record system and were trained on how to enter data correctly and accurately.
(2) Data processing: We used standardized data processing processes, including data cleaning, data transformation and data analysis. All steps had clear operational guidelines to ensure the consistency and accuracy of data processing. In addition, we conducted regular quality checks on the processed data.
(3) Data analysis: We used appropriate statistical methods for data analysis, and statistical software to ensure the accuracy of the analysis. All analysis steps were recorded to ensure repeatability of the analysis.

## Results

### Comparison of clinical data between two groups

Among the 118 patients, the median overall survival (OS) was 19 months, and the median disease-free survival (DFS) was 12 months. There were statistically significant differences in preoperative albumin, cholesterol, HDL, creatinine, and total bilirubin between the low MHR group and the high MHR group (all *p* < 0.05, Table 1).

**TABLE 1:** Comparison of perioperative conditions between low and high MHR group.

Clinical characteristics	Low MHR group	High MHR group	*p-value*
	(n = 61)	(n = 57)	
Age in year [mean ± SD]	63.2 ± 9.8	64.0 ± 10.1	0.669
Smoking history,n (%)	20 (32.8)	18 (31.6)	0.888
Diabetes,n (%)	16 (26.2)	15 (26.3)	0.992
Preoperative monocyte,×10^9^/L, [mean ± SD]	0.43 ± 0.14	0.48 ± 0.16	0.066
ALB,g/L, [median (IQR)]	36.5 (33.1, 44.5)	34.4 (31.1, 37.6)	0.007*
ALT,U/L, [median (IQR)]	82.0 (48.0, 144.0)	85.0 (33.0, 169.5)	0.963
TC,mmol/L, [median (IQR)]	4.7 (3.9, 5.7)	5.1 (4.3, 7.1)	0.022
HDL,mmol/L, [median (IQR)]	0.7 (0.6, 0.9)	0.4 (0.2, 0.7)	<0.001*
CREA, umol/L, [median (IQR)]	68.3 (57.3, 145.8)	144.2 (78.9, 156.1)	<0.001*
TB,umol/L, [median (IQR)]	90.0 (48.8, 166.2)	166.0 (91.1, 267.3)	0.001*
CEA,ng/mL, [median (IQR)]	2.0 (1.2, 4.0)	3.2 (1.5, 4.1)	0.222
CA19–9,U/mL, [median (IQR)]	102.9 (30.1, 423.7)	208.6 (47.7, 757.2)	0.171
Preoperative biliary drainage,n (%)	35 (57.4)	24 (42.1)	0.097
Preoperative bleeding, ml, [median (IQR)]	500.0 (400.0, 600.0)	600.0 (400.0, 800.0)	0.088
Intraoperative transfusion,ml, [median (IQR)]	0.0 (0.0, 800.0)	400.0 (0.0, 800.0)	0.296
Operation duration,h, [median (IQR)]	11.0 (9.0, 12.0)	10.0 (9.0, 12.5)	0.828
Tumor differentiation			0.295
low	15 (24.6)	19 (33.3)	
moderate/high	46 (75.4)	38 (66.7)	
Tumor size,cm, [median (IQR)]	3.0 (2.0, 4.0)	3.5 (3.0, 4.0)	0.383
Lymph node metastases,n (%)	40 (65.6)	39 (68.4)	0.742
Portal system invasion,n (%)	26 (42.6)	32 (56.1)	0.142
R0,n (%)	58 (95.1)	53 (93.0)	0.629
TNM stage,n (%)			0.578
I-II	41 (67.2)	41 (72.0)	
III	20 (32.8)	16 (28.0)	
Complication,n (%)	20 (32.8)	22 (38.6)	0.510
Chemotherapy,n (%)	39 (64.0)	31 (54.4)	0.291

TC, total cholesterol; HDL, high-density lipoprotein cholesterol; CREA, creatinine; ALB, albumin; ALT, alanine aminotransferase; TB, total bilirubin; CEA, carcino-embryonic antigen, CA19-9 cancer antigen 19-9. *p < 0.05.

### Prognosis comparison between two groups

**FIGURE 2:**
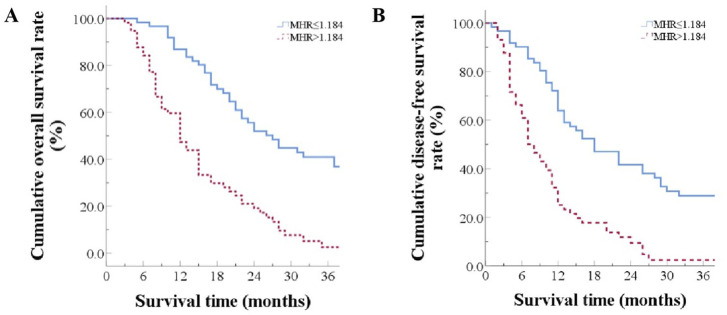
Prognosis Comparison Between Two Groups **(A)** Overall survival curves for patients grouped by high and low MHR; **(B)** Disease-free survival curves for patients grouped by high and low MHR.

The median overall survival time was 27.0 months in the low MHR group and 12.0 months in the high MHR group (χ2 = 30.575, *p* < 0.001, Table 2). The median recurrence-free survival time was 18.0 months in the low MHR group and 8.0 months in the high MHR group (χ2 = 26.330, *p* < 0.001) (Figure 2).

**TABLE 2:** Univariate & Multivariate analysis of risk factors for overall survival (OS).

Clinical characteristics (OS)	Univariate analysis			Multivariate analysis		
	*HR*	95%*CI*	*p-value*	*HR*	95%*CI*	*p-value*
Gender (Male/Female)	1.412	0.931-2.140	0.104			
Age (y)	1.009	0.989-1.028	0.387			
Smoking history (Yes/No)	1.343	0.879-2.050	0.173			
Diabetes (Yes/No)	1.039	0.658-1.640	0.869			
Leukocyte (×10^9^/L)	1.058	0.967-1.156	0.218			
Monocyte (×10^9^/L)	3.989	1.042-15.266	0.043*	1.638	0.310-8.659	0.561
Hemoglobin (g/L)	0.996	0.985-1.008	0.527			
Platelet (×10^9^/L)	1.001	0.998-1.003	0.573			
TC (mmol/L)	1.050	0.985-1.120	0.137			
HDL (mmol/L)	0.736	0.455-1.190	0.211			
MHR	1.201	1.087-1.327	<0.001*	1.225	1.074-1.397	0.002*
CREA (umol/L)	1.008	1.003-1.012	0.001*	1.008	1.003-1.013	0.002*
ALB (g/L)	1.015	0.970-1.063	0.516			
ALT (U/L)	1.000	0.998-1.003	0.829			
TB (umol/L)	1.001	0.999-1.003	0.295			
CEA (ng/mL)	1.001	0.999-1.004	0.31			
CA19-9 (U/mL)	1.000	1.000-1.000	0.153			
Preoperative biliary drainage (Yes/No)	1.176	0.789-1.753	0.426			
Preoperative bleeding (mL)	1.001	1.000-1.001	0.036*	1.000	0.999-1.000	0.615
Intraoperative transfusion (Yes/No)	1.000	1.000-1.001	0.106			
Operation duration (h)	1.182	1.089-1.282	<0.001*	1.173	1.058-1.300	0.002*
Tumor differentiation (Low/Moderate-High)	1.454	0.940-2.249	0.093	1.389	0.841-2.295	0.200
Tumor size (cm)	1.265	1.084-.1476	0.003*	1.151	0.970-1.366	0.106
Lymph node metastases (Yes/No)	1.422	0.922-2.193	0.111			
Portal system invasion (Yes/No)	1.464	0.981-2.184	0.062	1.104	0.676-1.802	0.692
Resection margin (R1/R0)	1.963	0.906-4.252	0.087	1.821	0.797-4.162	0.155
TNM stage (III/I-II)	1.819	1.189-2.781	0.006*	1.749	1.038-2.946	0.036*
Postoperative complication (Yes/No)	0.977	0.854-1.118	0.737			
Adjuvent chemotherapy (Yes/No)	0.760	0.508-1.137	0.182			

TC, total cholesterol; HDL, high-density lipoprotein cholesterol; MHR, monocyte to high-density lipoprotein ratio; CREA, creatinine; ALB, albumin; ALT, alanine aminotransferase; TB, total bilirubin; CEA, carcino-embryonic antigen; CA, 19-9 cancer antigen 19-9. *p < 0.05.

### Analysis of factors affecting overall survival

Univariate Cox regression analysis showed that preoperative monocyte count (HR = 3.989, 95% CI: 1.042-15.266, *p* = 0.043), preoperative MHR (HR = 1.201, 95% CI: 1.087-1.327, *p* < 0.001), preoperative creatinine (HR = 1.008, 95% CI: 1.003-1.012, *p* = 0.001), intraoperative blood loss (HR = 1.001, 95% CI: 1.000-1.001, *p* = 0.036), operation time (HR = 1.182, 95% CI: 1.089-1.282, *p* < 0.001), tumor size (HR = 1.265, 95% CI: 1.084-1.476, *p* = 0.003), portal vein system invasion (HR = 1.464, 95% CI: 0.981-2.184, *p* = 0.062), resection type (HR = 1.963, 95% CI: 0.906-4.252, *p* = 0.087), and TNM stage (HR = 1.819, 95% CI: 1.189-2.781, *p* = 0.006) were significantly associated with overall survival. Factors with *p* < 0.1 in univariate analysis were included in the Cox proportional hazards model for multivariate analysis, which revealed that preoperative MHR (HR = 1.225, 95% CI: 1.074-1.397, *p* = 0.002), preoperative creatinine (HR = 1.008, 95% CI: 1.003-1.013, *p* = 0.002), operation time (HR = 1.173, 95% CI: 1.058-1.300, *p* = 0.002), and TNM stage (HR = 1.749, 95% CI: 1.038-2.946, *p* = 0.036) were associated with poor prognosis (Table 2; Table 4).

### Analysis of factors affecting disease-free survival

Univariate Cox regression analysis showed that preoperative MHR (HR = 1.142, 95% CI: 1.035-1.259, *p* = 0.008), preoperative creatinine (HR = 1.006, 95% CI: 1.002-1.011, *p* = 0.005), operation time (HR = 1.124, 95% CI: 1.037-1.217, *p* = 0.004), tumor size (HR = 1.707, 95% CI: 1.106-2.634, *p* = 0.016), and TNM stage (HR = 1.720, 95% CI: 1.124-2.632, *p* = 0.012) were significantly associated with disease-free survival. Multivariate analysis of factors with *p* < 0.1 in univariate analysis showed that preoperative MHR (HR = 1.136, 95% CI: 1.003-1.287, *p* = 0.044), preoperative creatinine (HR = 1.006, 95% CI: 1.002-1.011, *p* = 0.009), and TNM stage (HR = 1.704, 95% CI: 1.028-2.827, *p* = 0.039) were associated with disease-free survival (Table 3; Table 4).

**TABLE 3:** Univariate & Multivariate analysis of risk factors for Disease-free Survival (DFS).

Clinical characteristics (RFS)	Univariate analysis			Multivariate analysis		
	*HR*	95%*CI*	*p-value*	*HR*	95%*CI*	*p-value*
Gender (Male/Female)	1.195	0.972-1.470	0.091	1.270	0.807-1.999	0.302
Age (y)	1.006	0.986-1.026	0.558			
Smoking history (Yes/No)	1.163	0.761-1.777	0.486			
Diabetes (Yes/No)	1.174	0.752-1.831	0.481			
Leukocyte (×10^9^/L)	1.039	0.946-1.141	0.422			
Monocyte (×10^9^/L)	3.014	0.819-11.088	0.097	1.276	0.269-6.044	0.759
Hemoglobin (g/L)	0.995	0.984-1.006	0.330			
Platelet (×10^9^/L)	1.000	0.998-1.003	0.837			
TC (mmol/L)	1.038	0.974-1.106	0.252			
HDL (mmol/L)	0.852	0.539-1.348	0.495			
MHR	1.142	1.035-1.259	0.008*	1.136	1.003-1.287	0.044*
CREA (umol/L)	1.006	1.002-1.011	0.005*	1.006	1.002-1.011	0.009*
ALB (g/L)	1.011	0.967-1.058	0.625			
ALT (U/L)	1.001	0.999-1.004	0.222			
TB (umol/L)	1.001	0.999-1.003	0.200			
CEA (ng/mL)	1.001	0.999-1.003	0.414			
CA19-9 (U/mL)	1.000	1.000-1.000	0.182			
Preoperative biliary drainage (Yes/No)	0.950	0.638-1.416	0.802			
Preoperative bleeding (mL)	1.000	1.000-1.001	0.092	1.000	0.999-1.000	0.600
Intraoperative transfusion (Yes/No)	1.000	1.000-1.001	0.211			
Operation duration (h)	1.124	1.037-1.217	0.004*	1.102	0.998-1.217	0.054
Tumor differentiation (Low/Moderate-High)	1.707	1.106-2.634	0.016*	1.558	0.963-2.521	0.071
Tumor size (cm)	1.266	1.083-1.480	0.003*	1.137	0.954-1.357	0.152
Lymph node metastases (Yes/No)	1.146	0.752-1.745	0.527			
Portal system invasion (Yes/No)	1.410	0.945-2.103	0.092	1.037	0.632-1.701	0.886
Resection Margin (R1/R0)	1.231	0.500-3.040	0.650			
TNM stage (III/I-II)	1.720	1.124-2.632	0.012*	1.704	1.028-2.827	0.039*
Postoperative complication (Yes/No)	0.890	0.588-1.348	0.583			
Postoperatvie chemotherapy (Yes/No)	0.888	0.594-1.329	0.565			

TC, total cholesterol; HDL, high-density lipoprotein cholesterol; MHR, monocyte to high-density lipoprotein ratio; CREA, creatinine; ALB, albumin; ALT, alanine aminotransferase; TB, total bilirubin; CEA, carcino-embryonic antigen; CA, 19-9 cancer antigen 19-9. *p < 0.05.

**TABLE 4:** Univariate & Multivariate analysis of risk factors for overall survival (OS) and Disease-free Survival (DFS) in patients.

Clinical characteristics	OS						DFS					
	Univariate analysis			Multivariate analysis			Univariate analysis			Multivariate analysis		
	*HR*	95%*CI*	*p-value*	*HR*	95%*CI*	*p-value*	*HR*	95%*CI*	*p-value*	*HR*	95%*CI*	*p-value*
Monocyte (×10^9^/L)	3.989	1.042-15.266	0.043*	1.638	0.310-8.659	0.561						
MHR	1.201	1.087-1.327	<0.001*	1.225	1.074-1.397	0.002*	1.142	1.035-1.259	0.008*	1.136	1.003-1.287	0.044*
CREA (umol/L)	1.008	1.003-1.012	0.001*	1.008	1.003-1.013	0.002*	1.006	1.002-1.011	0.005*	1.006	1.002-1.011	0.009*
Preoperative bleeding (mL)	1.001	1.000-1.001	0.036*	1.000	0.999-1.000	0.615						
Operation duration (h)	1.182	1.089-1.282	<0.001*	1.173	1.058-1.300	0.002*	1.124	1.037-1.217	0.004*	1.102	0.998-1.217	0.054
Tumor differentiation (Low/Moderate-High)							1.707	1.106-2.634	0.016*	1.558	0.963-2.521	0.071
Tumor size (cm)	1.265	1.084-.1476	0.003*	1.151	0.970-1.366	0.106	1.266	1.083-1.480	0.003*	1.137	0.954-1.357	0.152
TNM stage (III/I-II)	1.819	1.189-2.781	0.006*	1.749	1.038-2.946	0.036*	1.720	1.124-2.632	0.012*	1.704	1.028-2.827	0.039*

TC, total cholesterol; HDL, high-density lipoprotein cholesterol; MHR, monocyte to high-density lipoprotein ratio; CREA, creatinine; ALB, albumin; ALT, alanine aminotransferase; TB, total bilirubin; CEA, carcino-embryonic antigen; CA, 19-9 cancer antigen 19-9. *p < 0.05.

### Analysis of the predictive value of MHR in pancreatic cancer patients undergoing Radical resection with allogeneic vascular replacement

**FIGURE 3:**
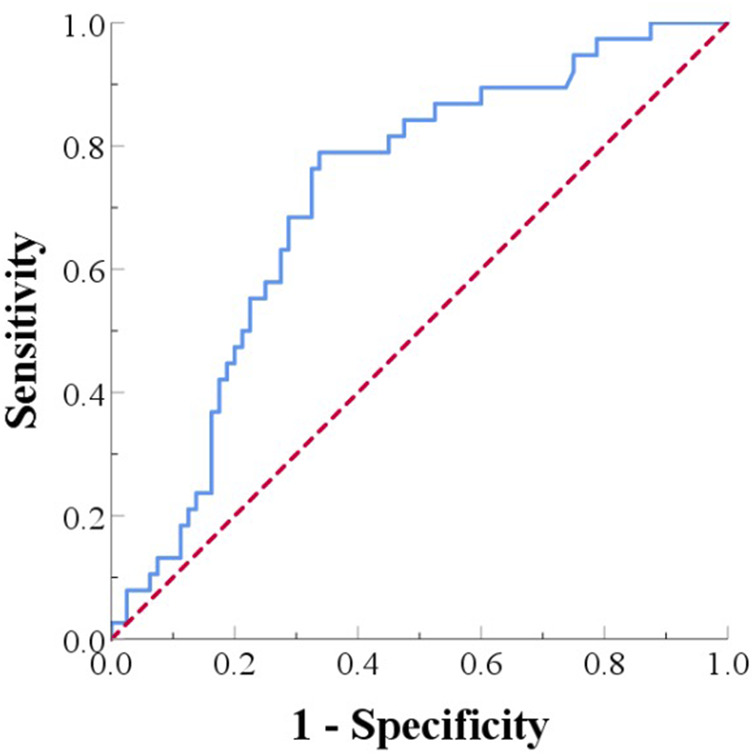
ROC curve for predicting 1-year survival after surgery in pancreatic cancer patients.

The receiver operating characteristic (ROC) curve was used to evaluate the predictive value of MHR for 1-year prognosis in PC patients after radical surgery. The area under the ROC curve (AUC) was used to assess the accuracy of the cutoff value. The ROC curve for predicting 1-year survival after radical resection and allogeneic vascular replacement in pancreatic cancer patients had an AUC of 0.714 (95% CI: 0.621-0.812). The optimal cutoff value was 1.184, with a sensitivity of 78.9% and a specificity of 66.2% for predicting 1-year survival (Figure 3).

### Development and validation of the prognostic prediction model nomogram

**FIGURE 4:**
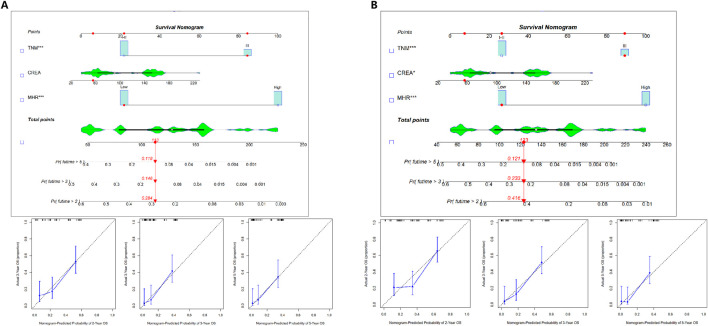
Prognostic prediction model nomogram and calibration curve.

OS and DFS prognostic prediction model nomograms were constructed using the entire cohort data. The final OS and DFS nomograms, incorporating TNM, CREA, and MHR, along with their calibration curve, are shown in Figure 4. These independent risk factors were used to predict the probability of OS.

## Discussion

In this study, we investigated the correlation between MHR and the prognosis of local advanced pancreatic cancer patients undergoing radical resection and allogeneic vascular replacement, and we reached the following conclusions: (Siegel et al., 2021): Preoperative MHR, as a novel biomarker, is an independent predictor of mortality and tumor recurrence in pancreatic cancer patients undergoing radical resection and allogeneic vascular replacement. Patients in the high MHR group had significantly lower overall survival (OS) and disease-free survival (DFS). (Huang et al., 2021). Multivariate analysis indicated that preoperative creatinine is also an independent predictor of postoperative mortality and tumor recurrence.

Studies have confirmed that monocytes have become important regulators of cancer development and progression (Olingy et al., 2019; Pushalkar et al., 2018), with their peripheral blood levels reflecting the severity of inflammation in both the systemic and tumor microenvironments (Ugel et al., 2021; Chatelain et al., 2024). In this study, peripheral monocytes were an independent risk factor for postoperative mortality in pancreatic cancer patients (*p* = 0.043). Monocytes are recruited to tumor sites via the CCL2/CCR2 axis, inhibiting CD8^+^ T cell infiltration and recruiting Tregs. Tregs in the tumor produce cytokines such as IL-4, IL-10, and IL-13, which cause monocytes to differentiate into tumor-associated macrophages (TAMs). Pro-angiogenic monocytes and TAMs in the tumor microenvironment can induce tumor angiogenesis by releasing angiogenic growth factors and pro-inflammatory cytokines and can also induce epithelial-mesenchymal transition (EMT) in pancreatic cancer cells, thereby promoting tumor growth and metastasis.

High-density lipoprotein (HDL), as an important lipoprotein, is considered a protective factor against pancreatic cancer. It has been shown to play a role in various cancers (von Eckardstein et al., 2023), including thyroid cancer, breast cancer, gastric cancer, and colorectal cancer (Samadi et al., 2019; Pirro et al., 2018). Dyslipidemia is increasingly recognized as an important mechanism in tumorigenesis, with the metastatic potential of tumor cells closely related to lipid-metabolizing enzymes. Low levels of HDL are associated with the development of distant metastases in tumors (Loosen et al., 2022). HDL can induce cholesterol depletion in TAMs, thereby weakening their tumor-promoting effects, enhancing the anti-inflammatory effects of neutrophils, promoting the activation of CD8^+^ and CD4^+^ T cells, and regulating the function of antigen-presenting cells and their complements (Smythies et al., 2010). Oberle R et al. also confirmed that HDL can mediate cholesterol depletion from tumor cells, leading to inhibition of pancreatic ductal adenocarcinoma (PDAC) cell growth and inducing tumor cell apoptosis. This effect is dependent on the action of SR-B1 and ABCA1 on tumor cells (Oberle et al., 2022).

The combination of monocytes and HDL represents a novel and comprehensive inflammatory marker of both injury and protection mechanisms, which may be more clinically valuable than monocyte and HDL levels alone. MHR is considered a biomarker for the extent of inflammation and oxidative stress. It has been proven to play a role in various tumors and is closely related to the occurrence, development, and prognosis of cancer. However, there have been no reports on the impact of MHR on pancreatic cancer patients. In this study, we confirmed that pancreatic cancer patients with high MHR had significantly shorter survival and tumor recurrence times, suggesting that preoperative MHR could serve as a potential biomarker for predicting postoperative prognosis in pancreatic cancer patients. ROC curve analysis indicated that when MHR >1.184, the risk of death within 1 year is high. Preoperative anti-inflammatory and lipid-lowering treatments, as well as early intervention to reduce MHR levels, may improve patient survival. Additionally, for patients with high MHR, timely postoperative adjustment of treatment and follow-up plans may also improve overall survival.

The level of MHR is positively correlated with the degree of tumor differentiation in colorectal cancer patients, and the tumors in patients with high MHR are more inclined to be less differentiated, which may be related to the tumor progression of colorectal cancer (Zhang et al., 2023). A history of diabetes is a common clinical risk factor in pancreatic cancer risk prediction models, and studies have shown that people with higher MHR may have more severe diabetes (Song et al., 2023), and poorly controlled diabetes increases the risk of pancreatic cancer in diabetic patients through the cellular proliferative effects of hyperglycemia, hyperinsulinemia, and abnormalities in insulin/IGF receptor pathways (Cui and Andersen Dana, 2012).

Local advanced PDAC represents a highly aggressive malignancy with a poor prognosis. Identifying reliable prognostic markers is crucial for tailoring treatment strategies and improving patient outcomes. One such marker that has garnered attention is the preoperative serum creatinine level. Elevated creatinine levels, indicative of renal impairment, have been associated with adverse outcomes in various cancers, including pancreatic cancer (Imamura et al., 2021). Our research demonstrated that elevated preoperative creatinine levels was an independent risk factor for poor prognosis in PDAC patients as well. Renal function is a fundamental aspect of systemic health, and elevated creatinine levels are a marker of impaired renal function. Chronic kidney disease (CKD) is known to be associated with increased mortality and morbidity in cancer patients. Impaired renal function can lead to the accumulation of metabolic waste products and toxins, which adversely affect overall physiological status and the body’s ability to tolerate and recover from aggressive cancer treatments such as surgery and chemotherapy. Studies have shown that CKD can exacerbate the inflammatory response and oxidative stress, further compromising patient outcomes (Zhuang et al., 2024). Meanwhile, Inflammation and nutritional status are critical determinants of cancer prognosis. Elevated creatinine levels often correlate with chronic inflammation and malnutrition, both of which are prevalent in PDAC patients. Chronic inflammation can promote tumor progression by inducing genetic mutations, stimulating angiogenesis, and suppressing antitumor immunity. Malnutrition, frequently observed in PDAC due to factors such as tumor-induced cachexia and impaired nutrient absorption, leads to a weakened immune response and reduced ability to withstand surgical and chemotherapeutic stress. Patients with elevated preoperative creatinine levels are at higher risk of developing postoperative complications, which can significantly impact their prognosis. Renal impairment is associated with an increased incidence of surgical site infections, delayed wound healing, and other perioperative complications. Additionally, renal dysfunction affects the pharmacokinetics of chemotherapeutic agents, necessitating dose adjustments that may reduce the efficacy of the treatment. This can limit therapeutic options and lead to suboptimal treatment outcomes.

Several studies have highlighted the prognostic significance of elevated preoperative creatinine levels in PDAC. A retrospective analysis of patients undergoing resection for PDAC found that those with higher creatinine levels had significantly shorter overall survival compared to those with normal renal function. Multivariate analysis confirmed that elevated creatinine was an independent predictor of poor prognosis, along with other established factors such as tumor stage and lymph node involvement (van Wijk et al., 2020). The recognition of elevated preoperative creatinine levels as an independent risk factor for poor prognosis in PDAC patients has significant clinical implications. It emphasizes the need for rigorous preoperative renal function assessment and highlights the potential benefits of nephroprotective strategies and early intervention for renal impairment. Additionally, understanding the interplay between renal function, inflammation, and nutritional status can guide the development of multidisciplinary treatment approaches that address these interconnected factors to improve patient outcomes.

However, this study has certain limitations. Firstly, it is a single-center retrospective cohort study with a small sample size, and large-scale multicenter studies are needed to confirm our findings. Secondly, some patients were lost to follow-up, which may introduce selection bias. Thirdly, due to the low survival rate of pancreatic cancer, we focused on the correlation between MHR and the 1-year postoperative survival rate of pancreatic cancer patients. Long-term follow-up results may be needed to explore the impact of MHR on long-term prognosis.

As one of the most prognostically poor malignancies, pancreatic cancer has become a global health issue with rising incidence rates, leading to early recurrence and poor outcomes for many patients. This study demonstrates that preoperative MHR is an independent predictor of OS and DFS in pancreatic cancer patients undergoing radical resection and allogeneic vascular replacement. This novel biomarker is simple, convenient, cost-effective, and easily obtainable, providing a reference for rational treatment planning. Early anti-inflammatory and lipid-lowering treatments targeting monocytes and HDL may open new avenues for improving patient prognosis and enhancing the quality of life for pancreatic cancer patients. However, research on the impact of MHR on pancreatic cancer progression and prognosis is still lacking, and the molecular mechanisms of MHR in tumor development and progression require further exploration and study.

## Conclusion

MHR, as an independent immune-inflammatory metabolic predictor of OS and DFS in patients with advanced PC after pancreaticoduodenectomy. Early monitoring and reduction of MHR may be of great significance in improving prognosis.

## Data Availability

The raw data supporting the conclusions of this article will be made available by the authors, without undue reservation.
